# Design, Synthesis, Molecular Modeling Studies and Biological Evaluation of N′-Arylidene-6-(benzyloxy)-4-oxo-1,4-dihydroquinoline-3-carbohydrazide Derivatives as Novel Anti-HCV Agents

**DOI:** 10.22037/ijpr.2019.112186.13586

**Published:** 2019

**Authors:** Sayyed Mohammad Ismaeil Mahboubi Rabbani, Rouhollah Vahabpour, Zahra Hajimahdi, Afshin Zarghi

**Affiliations:** a *Department of Pharmaceutical Chemistry, School of Pharmacy, Shahid Beheshti University of Medical Sciences, Tehran, Iran.*; b *Department of Medical Lab Technology, School of Allied Medical Sciences, Shahid Beheshti University of Medical Sciences, Tehran, Iran.*

**Keywords:** Synthesis, Design, 4-Oxo-1, 4-dihydroquinoline-3-carbohydrazide, HCV, NS5B polymerase, Molecular modeling studies

## Abstract

HCV-induced hepatitis is one of the most debilitating diseases. The limited number of anti-HCV drugs and drug-resistance necessitate developing of new scaffolds with different mode of actions. HCV non-structural protein 5B (NS5B) is an attractive target for development of novel inhibitors of HCV replication. In this paper, new N′-arylidene-6-(benzyloxy)-4-oxo-1,4-dihydroquinoline-3-carbohydrazide derivatives were designed based on the pharmacophores of HCV NS5B active site binding inhibitors. Designed compounds were synthesized and evaluated for their inhibitory activities in a cell-based HCV replicon system assay. Among tested compounds, compounds **18** and **20 **were found to be the most active (EC_50_ = 35 and 70 µM, respectively) with good selectivity index (SI > 2) in the corresponding series. Molecular modeling studies showed that the designed compounds are capable of forming key coordination with the two magnesium ions as well as interactions with other key residues at the active site of HCV NS5B.

## Introduction

Hepatitis C caused by the hepatitis C virus (HCV) has been considered as a leading cause for liver fibrosis, cirrhosis, and hepatocellular carcinoma (1, 2). According to the world health organization (WHO) report, chronic HCV infection has been estimated to affect about 71 million individuals worldwide (3). Prior to 2011, a combination of pegylated interferon alpha and ribavirin was used for HCV therapy, which sometimes caused severe adverse reactions, and was poorly tolerated (4). Currently, the treatment for HCV chronic infection consists of the addition of direct-acting antiviral drugs (DAAs) to the ribavirin regimen with or without pegylated interferon (5, 6). DAAs specifically target essential proteins in HCV life cycle including NS3 protease, NS5A serine protease, and NS5B RNA dependent RNA polymerase (7). In recent years, extensive efforts have been focused on discovery of direct-acting antiviral agents (8, 9). However, the emergence of drug resistance due to mutation of virus necessitate developing of new anti-HCV agents with different mode of actions and structures.

**Figure 1:**
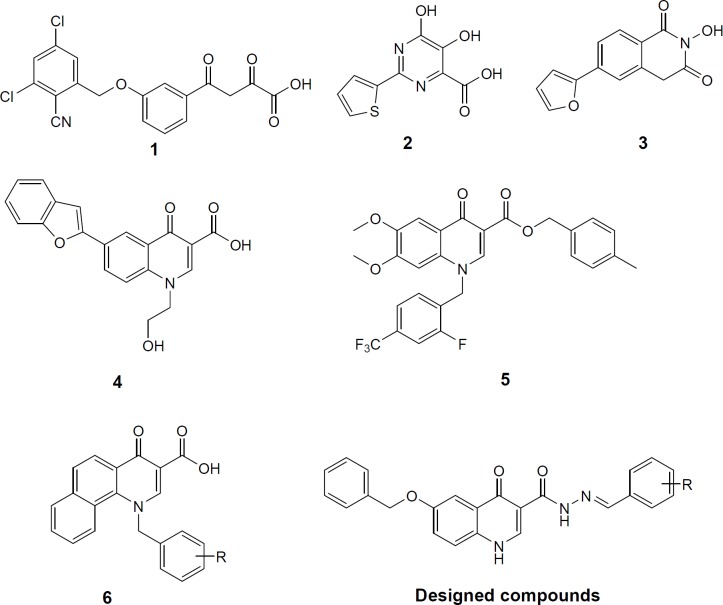
NS5B polymerase inhibitors (**1**, **2**, **3**, **4**, **5**, and **6**) and designed compounds

Non-structural protein 5B (NS5B) is a RNA-dependent RNA polymerase that plays a major role in the replicating the viral RNA of HCV. NS5B has no counterpart in human cells, and as a consequence, it is emerged as an attractive therapeutic target. Right-hand-like structure of NS5B polymerase consists of palm, thumb and fingers subdomains. Molecules that bind to and inhibit NS5B polymerase are classified into nucleoside inhibitors (NIs) that target active site and non-nucleoside inhibitors (NNIs) that target one of five allosteric sites referred as the thumb I, thumb II, palm I, palm II and palm-β (10, 11). The NS5B polymerase active site in the top region of the palm subdomain contains two Mg^2+^ or Mn^2+^ ions that are kept in their place by conserved residues Asp318, Asp319, and Asp220 (12). The metal ions play a critical role in the formation of the phosphodiester bond between sequential nucleotides (13). Therefore, compounds featuring chelating motif that bind to metal ions can be attributed to NS5B inhibition. This class of inhibitors represents a rare type of active site binding NNIs. Similar to NIs, active site binding NNIs have a high genetic barrier to development of drug resistance, because they bind to the highly conserved active site whereas typical NNIs bind to highly mutable allosteric sites (14-16). Active site binding NNIs inhibitors with a chelating motif generally also bear an aromatic ring directly connected to the chelating core. Since HCV NS5B polymerase shares a similar active site fold to HIV integrase (17), numerous HIV integrase inhibitors including diketoacids (compound** 1**), 5-hydroxy-pyrimidinones (compound **2**), 2-hydroxyisoquinoline-1,3-diones (compound **3**), quinolones (compounds **4**, **5**) have shown potent inhibition against HCV NS5B (Figure 1) (18-23). 

Quinolones are among the most privileged scaffolds in medicinal chemistry for designing inhibitors of interesting targets, a notable example being HIV IN inhibitor elvitegravir (24). We have previously introduced some quinolone derivatives based on HIV IN pharmacophores that effectively inhibited HIV-1 in cell culture (compound **6** as an example, Figure 1) (25-33). We expected that these derivatives could be HCV inhibitors through targeting NS5B. On this basis, we decided to retain quinolone scaffold as a main core while make changes on substituents on it. Previous studies focused on aromatic substitution on C-6 and C-7 of quinolone scaffold. This prompted us to design analogues with an aromatic substitution on other position of quinolone core. So we introduced arylidenehydrazide fragment at position 3. Since most of quinolone NS5B inhibitor possesses two hydrophobic moieties, we put a O-benzyl group at position C-6 of quinolone core. Combining the two structural modifications into a quinolone scaffold resulted in a new template. In this template, we explored the effect of various substituted benzylidenehydrazides on HCV inhibition.

## Experimental

*Chemistry*

All chemicals and solvents were purchased from “Merck/Sigma-Aldrich®” company and used directly without purification. Reaction progress was monitored by thin-layer chromatography (TLC) analysis on silicagel 60 F254 plates (Merck), visualized by UV light. Melting points (MP) were determined by the use of a Thomas–Hoover capillary apparatus. Infrared spectra were acquired using a Perkin-Elmer model 1420 spectrophotometer. A Bruker FT-400 MHz instrument (Bruker Biosciences, USA) was used to acquire ^1^H-NMR spectra. TMS was used as the internal standard and Chloroform-D and DMSO-d_6_ were used as the solvents. The amounts of coupling constant (J) are estimated in hertz (Hz) and spin multiples are reported as s (singlet), d (doublet), t (triplet), q (quartet) and m (multiplet). The mass spectroscopy experiments were performed by the use of a 6410 Agilent LCMS triple quadrupole mass spectrometer (LC-MS) with an electrospray ionization (ESI) interface. Microanalyses, determined for C and H, were within ± 0.4% of theoretical values.

*Synthesis of N-(4-(benzyloxy) phenyl)acetamide *(**8**) 

To a mixture of acetaminophen (1.0 g, 6.6 mmol) and sodium hydroxide (0.28 g, 6.6 mmol­) in methanol (10 mL­), benzyl bromide (0.8 mL, 6.6 mmol­) was added and stirred for 12 h at room temperature. Then, lots of water was added to the reaction mixture and the precipitate was filtered off and washed up by water and n-hexane give the compound **8**. Yield: 98%; mp: 120 °C; IR (KBr): υ (cm^-1^) 3276 (NH), 1656 (C=O), 1400-1600 (aromatic); GC-MS m/z: 241.0 (M, 100). 

*Synthesis of 4-(benzyloxy) aniline *(**9**) 

Compound **8** (1 g, 4 mmol­) was added to a 50% hydro-alcoholic solution of potassium hydroxide (KOH) and the mixture was refluxed for 48 hours. The mixture was poured into ice water and filtered to give the product **9**. Yield: 80%; mp: 45 °C; IR (KBr): υ (cm^-1^) 2837 - 3474 (NH_2_), 1400-1600 (aromatic); GC-MS m/z: 199.0 (M, 100). 

*Synthesis of diethyl 2-(((4-(benzyloxy)phenyl)amino)methylene)malonate *(**10**) 

Compound **9** (1 g, 5 mmol) was added to EMME (1 mL, 5 mmol­) and the mixture was stirred at room temperature for 10 minutes. After reaction completion, n-hexane was added to the mixture and the precipitate was filtered off to give the compound **10**. Yield: 99%; mp: 180 °C; IR (KBr): υ (cm^-1^) 1669 (C=O), 1400-1600 (aromatic); LC-MS (ESI) m/z 392 (M + 23). 

*Synthesis of ethyl 6-(benzyloxy)-4-oxo-1,4-dihydroquinoline-3-carboxylate *(**11**) 

A diphenyl ether solution of compound **10** (2 g, 5.4 mmol) containing catalytic 2-chlorobenzoic acid was heated by microwave irradiation (250 °C) for 5 minutes. After cooling, the reaction mixture was filtered and the precipitate was washed with n-hexane and water. Yield: 70%; mp: 210 °C; IR (KBr): υ (cm^-1^) 2725-3241 (NH), 1689 (C=O), 1400-1600 (aromatic); LC-MS (ESI) m/z 324.0 (M+1), 346.1 (M+23). 

*Synthesis of 6-(benzyloxy)-4-oxo-1,4-dihydroquinoline-3-carbohydrazide* (**12**) 

To a solution of compound **11** (2 g, 3.1 mmol) in absolute ethanol (10 mL), hydrazine hydrate (4 mL, 124 mmol) was added and the mixture was refluxed for 36 hours. After evaporation, the precipitated was filtered off and washed by water and n-hexane. Yield: 55%; mp: 65 °C; IR (KBr): υ (cm^-1^) 2601-3339 (NH), 1671 (C=O), 1400-1600 (aromatic); LC-MS (ESI) m/z: 310.0 (M+1). 

*General procedure for the synthesis of compounds* **13-25**

To a solution of compound **12** (0.5 g, 1.6 mmol) in absolute ethanol (10 mL), benzaldehyde derivatives (1.6 mmol) were added and the mixture was refluxed for 36 h. After cooling down to room temperature, the mixture was poured into ice water, and the precipitate was isolated by filtration. Target compounds were obtained by crystallization in ethanol.

*N′-Benzylidene-6-(benzyloxy)-4-oxo-1, 4-dihydroquinoline-3-carbohydrazide* (**13**)

Yield: 97%: white powder; mp: 130 ºC (decomposed), IR (KBr): υ (cm^-1^) 2725-3223 (NH), 1658 (C=O), 1450-1600 (aromatic C=C); LC-MS (ESI) m/z 398.0 (M+1, 100%); ^1^H-NMR (DMSO-*d*_6_, 400 MHz) δ ppm, 5.26 (s, 2H, CH_2_), 7.29-7.37 (3H, m, H_2_, H_4_, H_6_-benzyl), 7.40-7.44 (2H, t, H_3_, H_5_-benzyl, J = 8 Hz), 7.50-7.53 (3H, m, H_3_, H_4_, H_5_-benzylidene), 7.71-7.76 (3H, m, H_5_-quinolone/ H_2_, H_6_-benzylidene), 7.80-7.84 (2H, m, H_7_, H_8_-quinolone), 8.43 (1H, s, H-C=N-), 8.80 (1H, s, H_2_-quinolone), 12.97 (1H, br s, NH-amide), 13.50 (1H, s, NH-quinolone); ^13^C-NMR (DMSO-*d*_6_, 100 MHz) δ ppm, 21.53, 70.05, 106.62, 109.49, 122.11, 123.88, 127.59, 128.15, 128.41, 128.79, 128.98, 129.85, 130.00, 132.27, 137.16, 140.18, 143.95, 147.69, 156.22, 162.18, 175.47; Anal. Calcd for C_24_H_19_N_3_O_3_: C, 72.53; H, 4.82; N, 10.57. Found: C, 72.66; H, 4.59; N, 10.50.

*6-(Benzyloxy)-N′-(4-(methylthio)benzylidene)-4-oxo-1,4-dihydroquinoline-3-carbohydrazide* (**14**)

Yield: 90%; yellow powder; mp: 160 ºC (decomposed), IR (KBr): υ (cm^-1^) 2533-3513 (NH), 1653 (C=O), 1450-1600 (aromatic C=C); LC-MS (ESI) m/z: 444.0 (M+1, 100%), 909.3 (2M+23, 20%); ^1^H-NMR (DMSO-*d*_6_, 400 MHz) δ ppm, 2.56 (3H, s, CH_3_), 5.22 (2H, s, CH_2_), 7.23-7.26 (1H, q, H_7_-quinolone, *J*= 5 Hz/ 2 Hz), 7.35-7.41 (4H, m, H_3_, H_5_- benzylidene/ H_4_-benzyl/ NH-amide), 7.43-7.46 (2H, t, H_3_, H_5_-benzyl, *J*=7.6 Hz), 7.53-7.55 (2H, d, H_2_, H_6_-benzyl, *J*=7.4 Hz), 7.57-7.59 (1H, d, H_8_-quinolone, *J*=8.7 Hz), 7.70-7.72 (2H, d, H_2_, H_6_-benzylidene, *J*=8.3 Hz), 7.75-7.76 (1H, d, H_5_-quinolone, *J*=3 Hz), 8.30 (1H, d, H-C=N-, *J*=6 Hz), 8.78 (1H, s, H_2_-quinolone), 12.97 (1H, br s, NH-amide), 14.86 (1H, s, NH-quinolone); ^13^C-NMR (DMSO-*d*_6_, 100 MHz) δ ppm, 14.73, 70.07, 106.68, 109.58, 121.72, 124.04, 126.07, 127.52, 128.02, 128.16, 128.43, 128.98, 131.37, 134.53, 137.12, 141.27, 143.55, 147.44, 156.32, 161.99, 175.58; Anal. Calcd for C_25_H_21_N_3_O_3_S: C, 72.53; H, 4.82; N, 10.57. Found: C, 72.42; H, 4.56; N, 10.23.

*6-(Benzyloxy)-N′-(4-fluorobenzylidene)-4-oxo-1,4-dihydroquinoline-3-carbohydrazide* (**15**)

Yield: 85%; white powder; mp: 305 ºC (decomposed), IR (KBr): υ (cm^-1^) 2680-3444 (NH), 1662 (C=O), 1450-1600 (aromatic C=C); LC-MS (ESI) m/z 416.2 (M+1, 100%), 853.3 (2M+23, 71%); ^1^H-NMR (DMSO-*d*_6_, 400 MHz) δ ppm, 5.26 (2H, s, CH_2_), 7.29-7.37 (3H, m, H_2_, H_4_, H_6_-benzyl), 7.40-7.44 (2H, t, H_3_, H_5_-benzyl, J=8 Hz), 7.49-7.51 (3H, d, H_7_-quinolone/ H_2_, H_6_-fluorobenzylidene, J=8 Hz), 7.70-7.76 (1H, m, H5, H_8_-quinolone), 7.80-7.83 (2H, q, H_3_, H_5_-fluorobenzylidene, J=4 Hz), 8.42 (1H, s, H-C=N), 8.80 (1H, s, H_2_-quinolone), 12.98 (1H, br s, NH-amide), 13.55 (1H, s, NH-quinolone); ^13^C-NMR (DMSO-*d*_6_, 100 MHz) δ ppm 70.04, 106.61, 109.33, 116.20, 116.42, 122.42, 123.75, 127.62, 128.15, 128.40, 128.97, 129.66, 129.74, 131.62, 135.57, 137.18, 144.27, 146.50, 156.16, 162.25, 162.44, 164.71, 175.40; Anal. Calcd for C_24_H_18_FN_3_O_3_: C, 69.39; H, 4.37; N, 10.12. Found: C, 69.21; H, 4.12; N, 10.23. 

*6-(Benzyloxy)-N′-(4-chlorobenzylidene)-4-oxo-1,4-dihydroquinoline-3-carbohydrazide* (**16**)

Yield: 95%; white powder; mp: 329 ºC (decomposed), IR (KBr): υ (cm^-1^) 2676-3312 (NH), 1644 (C=O), 1450-1600 (aromatic C=C); LC-MS (ESI) m/z 432.0 (M+1, 100%), 454.0 (M+23, 36%), 885.0 (2M+23, 18%); ^1^H-NMR (DMSO-*d*_6_, 400 MHz) δ ppm, 5.26 (2H, s, CH_2_), 7.33-7.37 (1H, t, H_4_-benzyl, J= 8 Hz), 7.40-7.44 (2H, t, H_3_, H_5_-benzyl, J= 8 Hz), 7.49-7.54 (5H, m, H_7_-quinolone/H_2_, H_6_-chlorobenzylidene/ H_2_, H_6_-benzyl), 7.71-7.79 (4H, m, H_3_, H_5_-chlorobenzylidene/ H_5_, H_8_-quinolone), 8.43 (1H, s, H-C=N), 8.80 (1H, s, H_2_-quinolone), 12.98 (1H, br s, NH-amide), 13.49 (1H, s, NH-quinolone). ^13^C-NMR (DMSO-*d*_6_, 100 MHz) δ ppm 70.06, 106.67, 109.48, 121.66, 124.09, 127.51, 128.16, 128.43, 128.98, 129.21, 129.36, 129.56, 130.50, 133.06, 133.90, 134.42, 134.83, 136.50, 137.10, 143.58, 146.61, 156.35, 161.14, 162.12, 175.62; Anal. Calcd for C_24_H_18_ClN_3_O_3_: C, 66.75; H, 4.20; N, 9.73. Found: C, 66.56; H, 4.03; N, 9.99.

*6-(Benzyloxy)-N′-(4-methylbenzylidene)-4-oxo-1,4-dihydroquinoline-3-carbohydrazide* (**17**)

Yield: 98%; white powder; mp: 330 ºC (decomposed), IR (KBr): υ 2765-3204 (NH), 1647 (C=O), 1450-1600 (aromatic C=C); LC-MS (ESI) m/z: 412.2 (M+1, 100%), 845.4 (2M+23, 29%); ^1^H-NMR (DMSO-*d*_6_, 400 MHz) δ ppm, 2.36 (3H, s, CH_3_), 5.26 (2H, s, CH_2_), 7.27-7.29 (2H, d, H_3_, H_5_-benzylidene, *J*= 8 Hz), 7.33-7.37 (1H, t, H_4_-benzyl, *J*= 8 Hz), 7.40-7.44 (2H, t, H_3_, H_5_-benzyl, *J*= 8 Hz), 7.48-7.51 (3H, m, H_7_-quinolone/ H_2_, H_6_-benzyl), 7.65-7.67 (2H, d, H_2_, H_6_-benzylidene, *J*=8 Hz), 7.70-7.76 (2H, m, H_5_, H_8_-quinolone), 8.36 (1H, s, H-C=N), 8.80 (1H, s, H_2_-quinolone), 12.98 (1H, br s, NH-amide), 13.52 (1H, s, NH-quinolone). ^13^C-NMR (DMSO-*d*_6_, 100 MHz) δ ppm 19.0, 70.07, 106.68, 109.48, 116.22, 116.44, 121.86, 124.00, 127.54, 128.16, 128.43, 128.98, 129.70, 129.78, 131.59, 134.73, 137.13, 143.74, 146.72, 156.30, 162.17, 162.28, 164.74, 175.56; Anal. Calcd for C_25_H_21_N_3_O_3_: C, 72.98; H, 5.14; N, 10.21. Found: C, 72.65; H, 5.29; N, 10.53.

*6-(Benzyloxy)-N′-(4-methoxybenzylidene)-4-oxo-1,4-dihydroquinoline-3-carbohydrazide *(**18**)

Yield: 97%; white powder; mp: 154 ºC (decomposed), IR (KBr): υ (cm^-1^) 2643-3151 (NH), 1646 (C=O), 1450-1600 (aromatic C=C); LC-MS (ESI) m/z 428.1 (M+1, 100%), 450.1 (M+23, 33%); ^1^H-NMR (DMSO-*d*_6_, 400 MHz) δ ppm, 3.82 (3H, s, CH_3_), 5.22 (2H, s, CH_2_), 7.01-7.04 (2H, d, H_3_, H_5_-benzylidene, *J*= 10 Hz), 7.32-7.36 (3H, m, H_3_, H_4_, H_5_-benzyl), 7.40-7.44 (2H, t, H_2_, H_6_-benzyl, *J*=8 Hz), 7.49-7.51 (d, 2H, H_2_, H_6_-benzylidene, *J*=8 Hz), 7.60-7.62 (1H, d, H_7_-quinolone, *J*=8 Hz), 7.68-7.74 (2H, m, H_5_, H_8_-quinolone, *J*=8 Hz), 8.28 (1H, s, H-C=N), 8.76 (1H, s, H_2_-quinolone), 12.98 (1H, br s, NH-amide), 14.18 (1H, s, NH-quinolone). ^13^C-NMR (DMSO-*d*_6_, 100 MHz) δ ppm 55.84, 69.87, 114.69, 115.78, 122.66, 128.17, 128.29, 128.91, 129.66, 137.59, 145.17, 157.12, 158.35, 162.08, 175.03; Anal. Calcd for C_25_H_21_N_3_O_4_: C, 70.25; H, 4.95; N, 9.83. Found: C, 70.60; H, 4.88; N, 9.60.

*6-(Benzyloxy)-N′-(3-fluorobenzylidene)-4-oxo-1,4-dihydroquinoline-3-carbohydrazide *(**19**)

Yield: 75%; white powder; mp: 305 ºC (decomposed), IR (KBr): υ (cm^-1^) 2670-3254 (NH), 1658 (C=O), 1450-1600 (aromatic C=C); LC-MS (ESI) m/z 416.3 (M+1, 100%), 853.3 (2M+23, 30%); ^1^H-NMR (DMSO-*d*_6_, 400 MHz) δ ppm, 5.27 (2H, s, CH_2_), 7.26-7.30 (1H, m, H_4_-benzyl, *J*=8 Hz), 7.34-7.37 (1H, t, H_5_-fluorobenzylidene, *J*=8 Hz), 7.41-7.44 (2H, t, H_3_, H_5_-benzyl, *J*= 8 Hz), 7.50-7.56 (5H, m, H_2_, H_6_-benzyl/ H_2_, H_4_, H_6_-fluorobenzylidene), 7.60-7.62 (1H, d, H_7_-quinolone, *J*=8 Hz), 7.72 - 7.76 (2H, m, H_5_, H_8_-fluorobenzylidene), 8.45 (1H, s, H-C=N); 8.81 (1H, s, H_2_-quinolone), 12.98 (1H, br s, NH-amide), 13.56 (1H, s, NH-quinolone); ^13^C-NMR (DMSO-*d*_6_, 100 MHz) δ ppm 70.08, 106.70, 109.42, 113.44, 113.67, 117.05, 117.25, 121.73, 123.91, 124.08, 127.52, 128.16, 128.43, 128.98, 131.31, 131.39, 134.51, 137.10, 137.49, 137.57, 143.68, 146.57, 156.36, 161.62, 162.23, 164.05, 175.62; Anal. Calcd for C_24_H_18_FN_3_O_3_: C, 69.39; H, 4.37; N, 10.12. Found: C, 69.52; H, 4.56; N, 10.30.

*6-(Benzyloxy)-N′-(2-fluorobenzylidene)-4-oxo-1,4-dihydroquinoline-3-carbohydrazide *(**20**)

Yield: 70%; white powder; mp: 324 ºC (decomposed), IR (KBr): υ (cm^-1^) 2581-3527 (NH), 1659 (C=O), 1450-1600 (aromatic C=C); LC-MS (ESI) m/z 416.3 (M+1, 100%), 853.4 (2M+23, 30%); ^1^H-NMR (DMSO-*d*_6_, 400 MHz) δ ppm, 5.27 (2H, s, CH_2_), 7.30-7.37 (3H, m, H_2_, H_4_, H_6_-benzyl), 7.41-7.44 (2H, t, H_3_, H_5_-benzyl, *J*= 8 Hz/ *J*=4 Hz), 7.48-7.55 (4H, m, H_7_-quinolone/ H_4_, H_5_, H_6_-fluorobenzylidene), 7.73-7.77 (2H, m, H_5_, H_8_-quinolone), 7.93-7.97 (1H, t, H_3_-fluorobenzylidene, *J*= 8 Hz), 8.56 (1H, s, H-C=N), 8.81 (1H, s, H_2_-quinolone), 12.98 (1H, br s, NH-amide), 13.53 (1H, s, NH-quinolone); ^13^C-NMR (DMSO-*d*_6_, 100 MHz) δ ppm 70.08, 106.68, 109.42, 113.44, 113.67, 117.05, 117.25, 121.73, 123.91, 124.08, 127.52, 128.15, 128.43, 128.99, 131.31, 131.39, 134.51, 137.10, 137.49, 137.57, 143.68, 146.57, 156.36, 161.62, 162.23, 164.05, 175.60; Anal. Calcd for C_24_H_18_FN_3_O_3_: C, 69.39; H, 4.37; N, 10.12. Found: C, 69.12; H, 4.20; N, 10.33. 

*6-(Benzyloxy)-N′-(3-chlorobenzylidene)-4-oxo-1,4-dihydroquinoline-3-carbohydrazide *(**21**)

Yield: 70%; white powder; mp: 291 ºC (decomposed), IR (KBr): υ (cm^-1^) 2620-3396 (NH), 1690 (C=O), 1450-1600 (aromatic C=C); LC-MS (ESI) m/z 432.3 (M+1, 100%), 454.2 (M+23, 70%); ^1^H-NMR (DMSO-*d*_6_, 400 MHz) δ ppm, 5.27 (2H, s, CH_2_), 7.34-7.37 (1H, t, H_4_-benzyl, *J*= 8 Hz/ *J*=4 Hz), 7.41-7.44 (2H, t, H_3_, H_5_-benzyl, *J*=8 Hz), 7.50-7.55 (5H, m, H_2_, H_6_-benzyl/ H_5_, H_7_, H_8_-quinolone), 7.71-7.76 (3H, m, H_4_, H_5_, H_6_-chlorobenzylidene), 7.80 (1H, s, H_2_-chlorobenzylidene), 8.43 (1H, s, H-C=N), 8.81 (1H, s, H_2_-quinolone), 12.98 (1H, br s, NH-amide), 13.52 (1H, s, NH-quinolone); ^13^C-NMR (DMSO-*d*_6_, 100 MHz) δ ppm 70.01, 106.51, 108.93, 123.34, 126.10, 126.77, 127.75, 128.13, 128.96, 129.85, 131.16, 134.02, 137.29, 137.41, 145.34, 145.54, 155.94, 163.08, 175.14; Anal. Calcd for C_24_H_18_ClN_3_O_3_: C, 66.75; H, 4.20; N, 9.73. Found: C, 66.56; H, 4.33; N, 10.01.

*6-(Benzyloxy)-N′-(3-methylbenzylidene)-4-oxo-1,4-dihydroquinoline-3-carbohydrazide *(**22**)

Yield: 80%; white powder; mp: 310 ºC (decomposed), IR (KBr): υ (cm^-1^) 2661- 3354 (NH), 1661 (C=O), 1450-1600 (aromatic C=C); LC-MS (ESI) m/z 412.2 (M+1, 100%), 823.3 (2M+1, 20%); ^1^H-NMR (DMSO-*d*_6_, 400 MHz) δ ppm, 2.37 (3H, s, CH_3_), 5.27 (2H, s, CH_2_), 7.25-7.27 (1H, d, H_4_-benzylidene, *J*=8 Hz), 7.34-7.38 (2H, t, H_5_-benzylidene/ H_4_-benzyl, *J*= 8 Hz), 7.41-7.44 (2H, t, H_3_, H_5_-benzyl, *J*= 8 Hz), 7.50-7.56 (4H, m, H_7_-quinolone/ H_6_-benzylidene/ H_2_, H_6_-benzyl), 7.60 (1H, s, H_2_-benzylidene); 7.73 - 7.76 (2H, d, H_5_, H_8_-quinolone, *J*= 8 Hz/ *J*=4 Hz), 8.37 (1H, s, H-C=N); 8.81 (1H, s, H_2_-quinolone), 12.97 (1H, br s, NH-amide), 13.43 (1H, s, NH-quinolone); ^13^C-NMR (DMSO-*d*_6_, 100 MHz) δ ppm 21.40, 70.08, 106.68, 109.58, 121.66, 124.09, 125.15, 127.53, 127.78, 128.16, 128.43, 128.98, 129.15, 131.16, 134.45, 134.88, 137.11, 138.43, 143.55, 147.90, 156.35, 162.05, 175.61; Anal. Calcd for C_25_H_21_N_3_O_3_: C, 72.98; H, 5.14; N, 10.21. Found: C, 72.70; H, 5.32; N, 10.37.

*6-(Benzyloxy)-N′-(2-methylbenzylidene)-4-oxo-1,4-dihydroquinoline-3-carbohydrazide *(**23**)

Yield: 85%; white powder; mp: 290 ºC (decomposed), IR (KBr): υ (cm^-1^) 2563-3529 (NH), 1650 (C=O), 1450-1600 (aromatic C=C); LC-MS (ESI) m/z 412.2.0 (M+1, 100%), 823 (2M+1, 30%); ^1^H-NMR (DMSO-*d*_6_, 400 MHz) δ ppm, 2.50 (3H, s, CH_3_), 5.27 (2H, s, CH_2_), 7.25-7.37 (4H, m, H_3_-benzylidene/H_2_, H_4_, H_6_-benzyl), 7.41-7.44 (2H, t, H_3_, H_5_-benzyl, *J*= 8 Hz/ J= 4 Hz), 7.50-7.55 (3H, m, H_4_, H_5_, H_6_-benzylidene); 7.73-7.77 (2H, m, H_5_, H_7_-quinolone), 7.85-7.87 (1H, d, H_8_-quinolone, *J*= 8 Hz), 8.60 (1H, s, H-C=N), 8.81 (1H, s, H_2_-quinolone), 12.95 (1H, br s, NH-amide), 13.40 (1H, s, NH-quinolone); ^13^C-NMR (DMSO-*d*_6_, 100 MHz) δ ppm 19.64, 70.06, 106.65, 109.62, 121.71, 124.06, 126.55, 126.64, 127.56, 128.12, 128.42, 128.99, 130.11, 131.32, 132.91, 134.52, 137.10, 137.47, 143.60, 146.27, 156.33, 162.07, 175.55; Anal. Calcd for C_25_H_21_N_3_O_3_: C, 72.98; H, 5.14; N, 10.21. Found: C, 72.74; H, 5.41; N, 10.33.

*6-(Benzyloxy)-N′-(3-methoxybenzylidene)-4-oxo-1,4-dihydroquinoline-3-carbohydrazide *(**24**)

Yield: 70%; white powder; mp: 280 ºC (decomposed); IR (KBr), υ (cm^-1^) 2665-3424 (NH), 1663 (C=O), 1450-1600 (aromatic C=C); LC-MS (ESI) m/z 428.3 (M+1, 100%), 856.4 (2M+1, 14%); ^1^H-NMR (DMSO-*d*_6_, 400 MHz) δ ppm, 3.82 (3H, s, CH_3_), 5.27 (2H, s, CH_2_), 7.00-7.03 (1H, m, H_2_-benzylidene), 7.32-7.44 (6H, m, H_4_-benzylidene, H_2_, H_3_, H_4_, H_5_, H_6_-benzyl); 7.50 - 7.54 (3H, q, H_7_-quinolone/H_5_, H_6_-benzyl, *J*=8 Hz/ *J*=4 Hz), 7.72-7.76 (2H, m, H_5_, H_8_-quinolone, *J*=8 Hz/ *J*= 4 Hz), 8.39 (1H, s, H-C=N); 8.80 (1H, s, H_2_-quinolone), 12.95 (1H, br s, NH-amide), 13.51 (1H, s, NH-quinolone); ^13^C-NMR (DMSO-*d*_6_, 100 MHz) δ ppm 55.63, 70.07, 106.68, 109.54, 111.94, 116.52, 120.48, 121.67, 124.08, 127.52, 128.17, 128.43, 128.98, 130.35, 134.44, 136.34, 137.11, 143.55, 147.81, 156.35, 159.96, 162.08, 175.62; Anal. Calcd for C_25_H_21_N_3_O_4_: C, 70.25; H, 4.95; N, 9.83. Found: C, 70.01; H, 5.97; N, 9.68.

*6-(Benzyloxy)-N′-(2-methoxybenzylidene)-4-oxo-1,4-dihydroquinoline-3-carbohydrazide *(**25**)

Yield: 85%; white powder; mp: 299 ºC (decomposed), IR (KBr): υ (cm^-1^) 2481-3164 (NH), 1642 (C=O), 1450-1600 (aromatic C=C); LC-MS (ESI) m/z 428.3 (M+1, 100%), 877.4 (2M+23, 25%); ^1^H-NMR (DMSO-*d*_6_, 400 MHz) δ ppm, 3.90 (3H, s, CH_3_), 5.26 (2H, s, CH_2_), 7.02-7.06 (1H, t, H_5_-benzylidene, *J* = 8 Hz), 7.12-7.14 (1H, d, H_3_-benzylidene, *J* = 8 Hz), 7.34-7.38 (1H, t, H_4_-benzyl, *J* = 8 Hz), 7.41-7.46 (3H, m, H_3_, H_5_-benzyl/ H_4_-benzylidene), 7.50-7.53 (3H, m, H_2_, H_6_-benzyl/ H_6_-benzylidene, *J*=8 Hz/ *J*=4 Hz), 7.72-7.77 (2H, m, H_5_, H_7_-quinolone, *J*=8 Hz/ *J*=1 Hz), 7.86-7.88 (1H, m, H_8_-quinolone, *J* = 8 Hz/1 Hz), 8.57 (1H, s, H-C=N); 8.80 (1H, s, H_2_-quinolone), 12.95 (1H, s, NH-amide), 13.53 (1H, s, NH-quinolone); ^13^C-NMR (DMSO-*d*_6_, 100 MHz) δ ppm 56.22, 70.00, 106.45, 126.04, 128.14, 128.38, 128.97, 137.27, 155.94, 158.12, 175.30; Anal. Calcd for C_25_H_21_N_3_O_4_: C, 70.25; H, 4.95; N, 9.83. Found: C, 70.41; H, 5.11; N, 10.02.

*Molecular modeling study*

The active compound **21** was selected for docking studies against HCV NS5B. 1GX6 was used for binding mode analysis of NS5B inhibitory activity. All the compounds were built and subsequently optimized using the HyperChem 8.0 software (34). The protein structure was prepared for docking using AutoDock tools 1.5.6 from MGL Tools package (35). Docking was performed by Autodock VINA program (36). 

Co-crystallized ligand and all water molecules were removed from crystal protein while manganese ions (Mn^2+^) at the active site of HCV NS5B were remained. Polar hydrogens were added and non-polar hydrogens were merged, finally, Kollman united atom charge and atom type parameter was added to 1GX6. Grid map dimensions (20×20×20) were set surrounding active site. 

*Cell Culture and virus production*

Huh7.5 cells originated from hepatocellular carcinoma which has been shown to support efficient HCV replication and production (kindly provided by Rice C) was cultured in Dulbecco′s modified Eagle′s medium (DMEM) supplemented with 10% fetal bovine serum (FBS), penicillin (100 U/mL) and streptomycin (100 μg/mL). 

The plasmid encoding the full genome of Japonicum Fulminant Hepatitis virus serotype 1 (JFH-1) strain (kindly provided by Wakita T) was used for *in-vitro* transcription to produce approximately high titer stocks of cell culture-produced virus (HCVcc) as previously described (37).

*RT-qPCR assay*

Huh7.5 cells were seeded into 48-well plates at a density of 3×10^5^ cells per well with DMEM containing 10% FBS. After 24 h, huh7.5 cells were infected with HCV viral stock (105 IU/mL) for 3 h, at which point the infection was stopped and each well was washed three times by sterile PBS to removed non-penetrated virions and then the fresh medium was added to each well. Subsequently, the infected cells were treated with newly synthesized compounds at different concentrations and the sofosbuvir (Sigma-Aldrich, USA) as a positive control. After three days inoculation, the supernatant medium was collected and the viral RNA was extracted using viral QIAamp viral RNA mini kit (Qiagen, Düsseldorf, Germany), according to the manufacturer protocol. Then, the amount of HCV replicon RNA was determined using Quantitative RT-PCR assay by hepatitis C virus viral load kit (Gene Proof, Czech Republic, with CE, IVD) according to the manufacturer’s instruction. 

The 50% effective concentration (EC50) was determined as the ratio of the normalized HCV RNA amount in treated samples relative to the controls (the samples without compound treatment) using GraphPad Prism5.0 software (GraphPad Software, San Diego, CA). 

*Cytotoxicity Assay*

The cytotoxicity of compounds to Huh-7.5 cells was measured in parallel with the HCV replication assay based on the cleavage of the yellow tetrazolium salt XTT® (sodium 3-[1 (phenylaminocarbonyl)-3, 4-tetrazolium]-bis(4-methoxy-6-nitro)benzene sulfonic acid) to generate an orange formazan dye by metabolic function of alive and active cells. Cells were treated with various concentrations (500, 250, 100, 25, and 12 μm/mL) of compounds and incubated for 72 hours. 

The cell cytotoxicity was determined using XTT proliferation assay kit (Sigma-Aldrich) following the manufacturer’s instructions. The 50% cytotoxic concentration (CC50) was defined as the concentration of compound that reduced cell proliferation by 50%.

## Results and Discussion

*Chemistry*

**Scheme 1:**
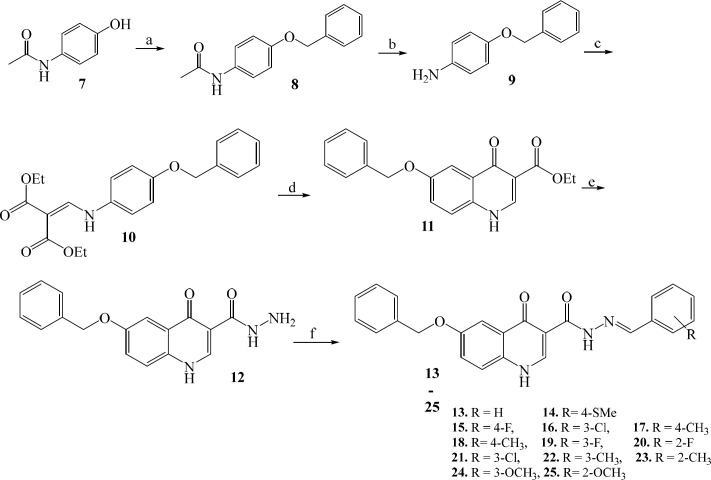
**Reagents and conditions:** (a) benzyl bromide, NaOH, CH_3_OH, rt, 12 h; (b) 50% KOH hydroalcoholic solution, reflux, 48 h; (c) EMME, rt, 10 min; (d) 2-chlorobenzoic acid, Ph_2_O, 250^ ○^C, MW, 5 min; (e) NH_2_NH_2_.H_2_O, absolute ethanol, reflux, 36 h; (f) benzaldehyde derivatives, absolute ethanol, reflux, 36 h

Target compounds **13-25** were prepared in good yields (70-98%) from the acetaminophen as a starting material as shown in scheme 1. Starting from benzylation of acetaminophen (**7**) with benzyl bromide followed by basic hydrolysis of acetamide group gave the *O*-benzyl aniline (**9**). The key quinolone intermediate (**11**) was synthesized via the well-known Gould–Jacobs approach (38). This procedure began with condensation of *O*-benzyl aniline (**9**) with ethoxymethylenemalonate diethyl ester (EMME) to produce malonate intermediate (**10**), which upon intramolecular cyclization afforded the ethyl quinolone-3-carboxylate (**11**). The resulting ester was converted to carbohydrazide analogue (**12**) via reaction with hydrazine hydrate. N′-Arylidene fragment in target compounds (**13-25**) were introduced through reaction of carbohydrazide intermediate **12** with substituted benzaldehydes. The structure of the synthesized compounds was confirmed by IR, ^1^H-NMR, and ESI-MS.

*HCV replicon assay*

Target compounds **13-25** were evaluated in a cell-based viral replication surrogate assay known as the replicon system (39). We hypothesized that our quinolone analogues will inhibit NS5B, likely via binding to the active site. To test this hypothesis, FDA-approved active site binding NI, sofosbuvir, was used as a control compound. In parallel, compounds were also evaluated for their cytotoxic effects in the same cell line to ensure that the activity is completely devoid of cytotoxicity effects. The biological results are expressed as EC_50_, CC_50_ and SI (selectivity index, given by the CC_50_/EC_50_ ratio).

As shown in Table 1, some of the target compounds were found active against HCV with the EC_50_ values in the range from 35 to 120 µM. Furthermore, except for compound **24**, all of these active compounds showed SI > 2, rendering them suitable as an inhibitor scaffold for HCV. The anti-HCV assay results revealed a few discernible structure-activity relationship (SAR) trends. First of all, introduction a suitable substituent on the phenyl ring of compounds was found to greatly impact potency as compound **13** with an unsubstituted phenyl was inactive (EC50>200 µM) whereas a number of analogues with substituted phenyl showed significant activity (compounds **20**-**24**). Secondly, the identity of substituent also appeared crucial for inhibition as among active compounds, anti-HCV activity decreased in the following order: Cl > Me > F > OMe > SMe. Thirdly, position of substituent on the phenyl ring influenced the anti-HCV activity as for nearly all substituents, *ortho*- or *meta*-position of the phenyl ring was tolerated better than *para*-position. Notably, compound **21** with Cl group in *meta*-position (EC_50_ = 35 µM) was proven to be considerably more potent than its *para*-counterpart (**16**, EC_50_ > 200 µM). In compounds with methyl substitution, changing from *ortho*- to *meta*-position increased the both activity (EC_50_ = 70 and 45 µM, respectively) and cytotoxicity (CC_50_ = 160 and 45 µM, respectively). Regarding the similarity of EC_50_ and CC_50_ of compound **22**, it seems that its anti-HCV activity was result of cytotoxicity. Compounds **20** and **24 **having, respectively, 2-fluoro and 3-methoxy substitutions displayed similar activities with an EC_50_ = 120 µM. These differences in activity and toxicity between substituents and their positions may reflect a fundamental change in their binding mode to the protein target or physicochemical properties, although further investigation is required to determine the structure–activity relationships in detail.

Overall, the best activities were observed with 3-chloro (compound **21**, EC_50_ = 35 µM) and 2-methyl (compound **23**, EC_50_ = 70 µM) substituents, suggesting that designed scaffold has the potential for antiviral discovery against HCV. Further optimization may be required to address the potency of compounds.

**Table 1:** EC50, CC50 and TI for newly - synthesized compounds

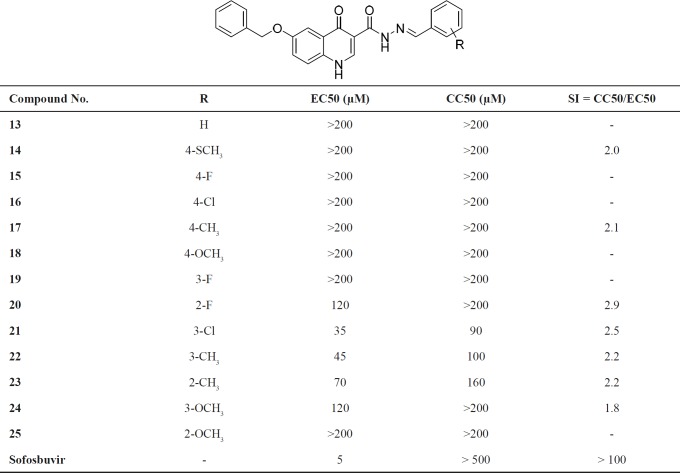

**Figure 2:**
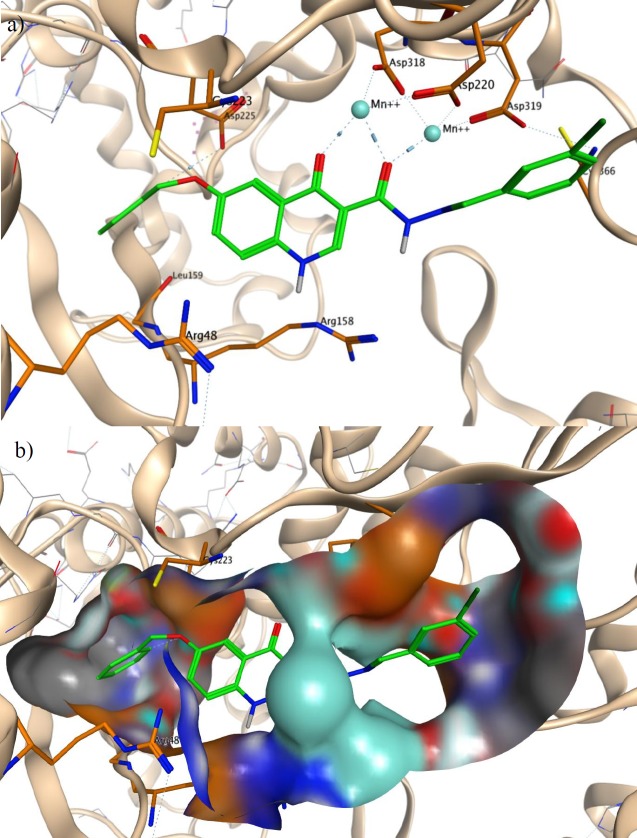
a Binding mode of compound **21** in the active site of HCV NS5B polymerase(PDB code: 1GX6). b Interaction of compound **21 ** with the surface of NS5B active site

*Molecular Modeling Study*

Our compounds were designed based on pharmacophore model of NS5B active site inhibitors. Therefore, we generated a docking model to investigate binding modes of designed compounds with NS5B active site. Docking model was generated on the basis of the existing X-ray crystallographic structure of NS5B polymerase complexed with uridine triphosphate (UTP) and divalent metal ions (PDB code: 1GX6) (40). The most active compound observed in anti-HCV assay, compound** 21** was docked into the binding pocket of NS5B active site after the removal of the original UTP. As shown in Figure 4, compound **21 **interacted with two Mn^2+^ that are coordinated by active site residues Asp220, Asp318 and Asp319. The Mn^2+ ^ions were chelated through two carbonyl groups of quinolone scaffold with the distances of 2.76, 2.37 and 2.42 Å. The O-benzyl moiety of compound **21** was inserted into a hydrophobic pocket in the enzyme consisting of Leu159 and Cys223. Leu159 is an important residue in interaction with the pyrimidine base of co-crystalized UTP. Oxygen atom of O-benzyl formed a hydrogen bond to the side chain of Asp225 with the distance of 3.76 Å, the same residue that is bonded to 2′-hydroxy group of UTP. In addition, NH group of quinolone scaffold formed interactions with side chain of Arg158 and Arg48. An extra hydrophobic bond was found between 3-chlorophenyl group of compound **21** and Cys366. These observed interactions are in agreement with reported studies on docking of NS5B active site inhibitors (41-43). So anti-HCV activity of synthesized compounds may be due to NS5B inhibition.

## Conclusion

In this study, in order to identify new scaffold as anti-HCV agents, we designed and synthesized new N′-arylidene-6-(benzyloxy)-4-oxo-1,4-dihydroquinoline-3-carbohydrazide derivatives. Results of cell-based HCV replicon assay revealed that several compounds (**20**, **21**, **22**, **23**, and **24**) exhibited moderate to good activity with EC_50_ values ranging from 35 to 120 µM. Molecular modeling studies suggested that the designed compounds might interact with two magnesium ions and other key residues in the NS5B polymerase active site. These results make the designed scaffold a promising starting point for development of more active compounds in future.
